# A microRNA signature and TGF-β1 response were identified as the key master regulators for spaceflight response

**DOI:** 10.1371/journal.pone.0199621

**Published:** 2018-07-25

**Authors:** Afshin Beheshti, Shayoni Ray, Homer Fogle, Daniel Berrios, Sylvain V. Costes

**Affiliations:** 1 WYLE, NASA Ames Research Center, Moffett Field, California, United States of America; 2 USRA, NASA Ames Research Center, Moffett Field, California, United States of America; 3 NASA Ames Research Center, Space Biosciences Division, Moffett Field, California, United States of America; University of Massachusetts Medical School, UNITED STATES

## Abstract

Translating fundamental biological discoveries from NASA Space Biology program into health risk from space flights has been an ongoing challenge. We propose to use NASA GeneLab database to gain new knowledge on potential systemic responses to space. Unbiased systems biology analysis of transcriptomic data from seven different rodent datasets reveals for the first time the existence of potential “master regulators” coordinating a systemic response to microgravity and/or space radiation with TGF-β1 being the most common regulator. We hypothesized the space environment leads to the release of biomolecules circulating inside the blood stream. Through datamining we identified 13 candidate microRNAs (miRNA) which are common in all studies and directly interact with TGF-β1 that can be potential circulating factors impacting space biology. This study exemplifies the utility of the GeneLab data repository to aid in the process of performing novel hypothesis–based research.

## Introduction

The spaceflight environment has been evaluated for human health risks due to hazardous factors such as ionizing radiation, microgravity, hypoxia, hypothermia as well as other associated physiological/psychological stressors. Space radiation and microgravity have been considered as the primary potential show-stoppers for long duration space exploration missions beyond LEO (Low Earth Orbit) and are thought to be driving many of the responses observed in flown organisms. Due to the high cost and limited availability of larger cohort for spaceflight samples, rodents have emerged as the primary choice among the model organisms for flight experiments. This is due to its similarity to the human genome [1] and their relative small size compared to other rodents. Omics data related to these studies have been made available to the public through NASA’s GeneLab platform (genelab.nasa.gov). GeneLab is an open repository that houses fully coordinated and curated experimental results, raw data and metadata pertaining to model organisms onboard International Space Station (ISS), Space Transportation Systems (STS), or the russian animal carry capsule called Bion-M1 [2]. GeneLab also houses ground NASA studies which model spaceflight environment. The vast amounts of genomic, proteomic, transcriptomic and metabolomic data from several organisms and cell culture experiments are available to researchers across the globe for *in silico* analysis and generating hypothesis-driven future research.

Several mice studies have been conducted in the past to assess the organ-specific physiological responses to microgravity in short-term and long-term space missions. X.W. Mao et.al. investigated the effect of a 13-day mission on the cutaneous tissue and found numerous genes encoding anti-oxidants and extra-cellular matrix (ECM) proteases along with genes responsible for reactive oxygen species (ROS) generation and gluconeogenesis to be upregulated in the space flown mice [3]. Spaceflight conditions have also been shown to exert deleterious effects on the musculoskeletal system. The short-term spaceflight response in the gastrocnemius muscle included decreased PI3-kinase/Akt/mTOR signaling, myogenic cell proliferation, and differentiation [4]. The long-term responses in the calf *soleus* identified changes in expression of genes involved in maintaining calcium homeostasis, supporting contractile machinery, muscle development, cell metabolism, and decreasing inflammatory and oxidative stress response [1]. Hepatic lipid metabolism was also evaluated in a 13.5-day mission and early signs of liver injury was detected in mice [5]. Although these previous studies are starting to reveal the biological impact of microgravity and/or space radiation on single components of the host, the overall global response on the host has not yet been determined.

Analysis of complex systems have indicated the difficulty in interpreting collective emergent behaviors occurring as a result of interaction between various components through separate analyses of those components [6]. Hence in this study, we provide a systems-level analyses to assess effects of spaceflight in rodents flown in different habitats for varying time-points, by evaluating the transcriptional changes in several tissues harvested either post-flight or on-orbit. Through an established systems biology approach [7–9] we identified a master regulator, TGF-β1, coordinating systemic responses to microgravity, at multiple biological scales. We were further able to predict that the global host response between the multiple linked tissues based on TGF-β1 was driven by circulating microRNAs (miRNAs). MiRNAs are small non-coding RNA, which can impact a large number of genes, proteins and DNA [10]. Due to the small size of miRNAs it has been found to be stably circulating throughout the blood, both free floating and encapsulated in exosomes [11]. We were able to predict that a spaceflight specific circulating miRNA signature has the potential to drive systemic TGF-β1 response in the host and display subsequent impact on health, calculated from a biological “health risk score”. The genes and miRNAs identified from our analyses can be targeted for future research involving efficient countermeasure design. Our study epitomizes the value of GeneLab data repository, which can be used not only to retrieve spaceflight data to assess organ/tissue-specific perturbations in molecular signaling networks, but also to establish the foundation of novel hypothesis–based spaceflight research aimed at characterizing the global impact of environmental stressors at multiple biological scales.

## Materials and methods

### Ethical approval

This study was conducted in accordance with all ethical standards.

### Data from GeneLab platform

**Fig 1:**
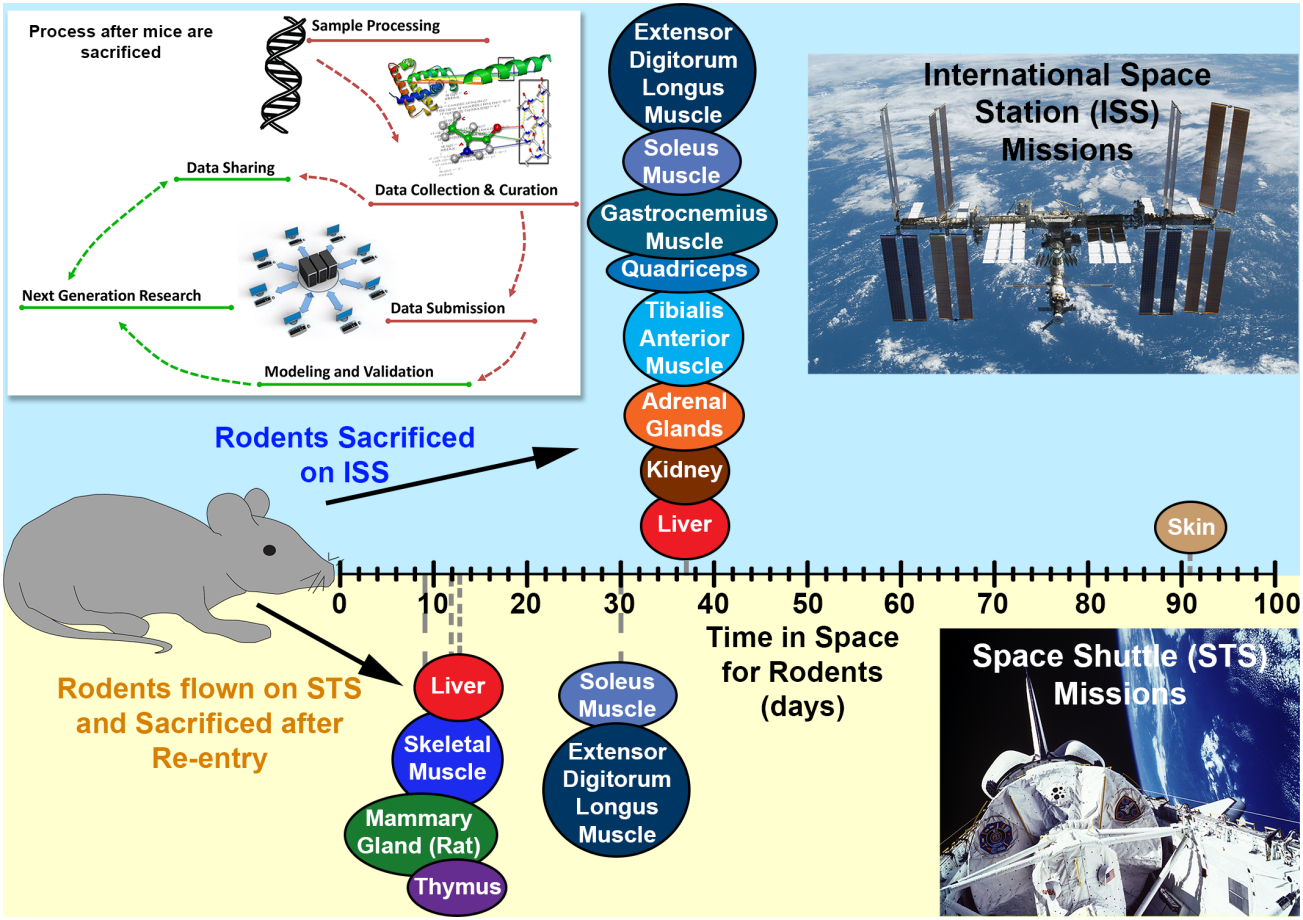
Illustration of methodology and tissues used for analysis. A schematic representation of the process flow in obtaining the omics dataset from GeneLab database from the tissues used for analyses in this manuscript is shown on the top left. The different tissues have been plotted along an x-axis for the amount of the time the rodents were in space before sacrificing. The upper panel (blue) represents tissues from rodents on the international space station (ISS) and the lower panel (yellow) represents the tissues from rodents on space shuttle missions (STS).

All data used for this manuscript were obtained from GeneLab (genelab.nasa.gov). The following datasets were used: GLDS-25, -21, -63, -111, -4, -61 and 48. Spaceflight mission and experimental details for each dataset such as, the handling of the rodents, tissue processing, RNA extraction and raw data pertaining to either microarray or RNA-sequencing, can be found in the GeneLab database. Briefly, we used 7 different murine and rat datasets for our analysis and examined the following tissues: liver, kidney, adrenal gland, thymus, mammary gland, skin, and skeletal muscle (soleus, extensor digitorum longus, tibialis anterior, quadriceps, and gastrocnemius) (Fig 1). Specific Details regarding these rodents during spaceflight are available in the supplemental materials and methods section.

### Transcriptome analysis

The available transcriptomic data for tissues from GLDS-25, -21, -63, -111, -4, -61 datasets were previously performed on different versions of Affymetrix platforms. The transcriptomic data for the tissues from the GLDS-48, -98, -101, -102, -103, -104, -105 datasets was processed using RNA-sequencing. Due to the incompatibility of processed data from these different platforms, all datasets were analyzed independently and the processed data was compared across all tissues. For the GLDS-25 and -4 datasets raw data was background adjusted and quantile normalized done using GenePattern [12]. For GLDS-21, -63, -111, and -61 datasets were background adjusted and quantile normalized using RMAExpress [13]. The probes from the pre-processed data were median collapsed using GenePattern. All tissue for the GLDS-48, -98, -101, -102, -103, -104, -105 data was pre-processed by having Illumina reads trimmed for sequencing adapters and phred quality score of 20 with Trim-Galore. Alignment to Gencode Release M15 (GRCm38.p5) transcript sequences and quantification of Gencode comprehensive gene annotations was performed with Kallisto (Kmer length = 31, bootstrap sequences = 100, paired-end, strand specific first read forward) [14]. Read and mapping quality was evaluated with RseQC [15]. Transcript and gene estimated counts and transcripts per million normalizations were performed with Sleuth [16]. All the data for each tissue/dataset was imported into MultiExperiment Viewer [17] and statistically significant genes was determined either by t-test with a p-value ≤ 0.05 or False Discovery Rate (FDR) ≤ 0.05 depending on statistical stringency needed to produce a reasonable number of genes to take forward for the rest of the higher-order analysis.

Pathway analysis and subsequent predictions in each tissue were done using the statistically significant genes with a fold-change ≥1.2 (or ≤-1.2) comparing Flight Conditions versus AEM ground controls. Ingenuity Pathway Analysis (IPA) software (Ingenuity Systems) was used to predict statistically significant activation or inhibition of upstream regulators, canonical pathways, biofunctions, and toxic functions using activation Z-score statistics (≥ 2, activated or ≤ −2, inhibited) [18]. Gene set enrichment analysis (GSEA) was done using the C2, C5, and Hallmarks gene sets with a FDR ≤ 0.05 from the entire list of genes and additional leading edge analysis was performed as described by Subramanian et. al [19]. All heat maps and principle component analysis (PCA) plots were generated using packages available through R (pheatmap for heat maps and scatterplot3d for PCA plots).

### Determination of key genes/drivers

We used a previously established unbiased systems biology method to determine key genes/drivers [7, 9, 20, 21] for each tissue. Briefly, this was done by determining the overlapping genes involved in the predictions made through IPA’s upstream regulators, canonical pathways, biofunctions and GSEA gene sets (C2, C5, and Hallmarks gene sets). The common genes identified through these statistically significant predictions can be thought of as the central drivers for the experiment being studied, since the absence of the genes will make these predictions null. To determine the key gene, which has the highest number of connections and can be thought of as the central hub for the set of key genes, we utilized IPA to define the total number of connections between all the key genes. Next, we plotted the genes using the radial plot tool which places the most connected gene in the center of the plot. Previous work involving similar statistically identified key genes provided experimental validation for this methodology using Western blots, qPCR, and other functional assays [7, 20].

### MicroRNA (miRNA) predictions and health risk score calculation

Through the use of p-value and activation Z-score statistics in IPA, the top 13 miRNAs impacting the key genes were determined. The upstream regulator tool in IPA was used to determine the top 10 statistically relevant miRNAs (p-values ≤ 1.17 × 10^−8^) and the prediction of activation/inhibition of all miRNAs was performed with activation Z-score ≥ 2 or ≤ −2 (with corresponding p-values ≤ 1.44 × 10^−5^). Activation Z-score statistics provides the actual functional activity for the miRNAs, which provides a more meaningful biological representation of the impact of the miRNAs than p-value alone. The activation Z-score analysis resulted in 4 miRNAs, with one miRNA overlapping with the top 10 p-value determined miRNAs. The activity of the remaining 9 miRNAs was determined through activation Z-score statistics in IPA. A full list of all predicted miRNAs can be found in S1 Table.

The “health risk score” (HRS) was determined by first associating the overall general impact on health for each miRNA from the literature. Then the HRS was calculated by subtracting the activation Z-score values (used to determine amount of activation or inhibition) of the miRNAs that provided a negative health impact from the miRNAs that provided a positive health impact. If the overall HRS is positive then it will indicate a beneficial impact on health, while a negative value will indicate a negative impact on health.

## Results and discussion

### Description of datasets utilized from GeneLab

GeneLab database was used to obtain microarray and RNA sequencing data pertaining to 12 different mouse datasets (GLDS-25, -21, -111, -4, -61, -48, -98, -101, -102, -103, -104, and -105) and one rat dataset (GLDS-63 –mammary gland). While microarray expression data was used for GLDS-25, -21, -63, -111, -4, -61 data sets, NGS (RNA-seq) data was utilized for all tissues related to GLDS-48, -98, -101, -102, -103, -104, -105 data sets. Several tissues were characterized in these datasets: liver, kidney, adrenal gland, thymus, mammary gland, skin, and skeletal muscle (soleus, extensor digitorum longus, tibialis anterior, quadriceps, and gastrocnemius). Fig 1 outlines the various tissues as well as the three different flight systems—five STS, two ISS and one BION-M1 biosatellite (BF) flights with their respective flight durations. The detailed description of the age, sex and strain of the animals and type of tissue harvested for each experiment can be found in the Methods section.

**Fig 2:**
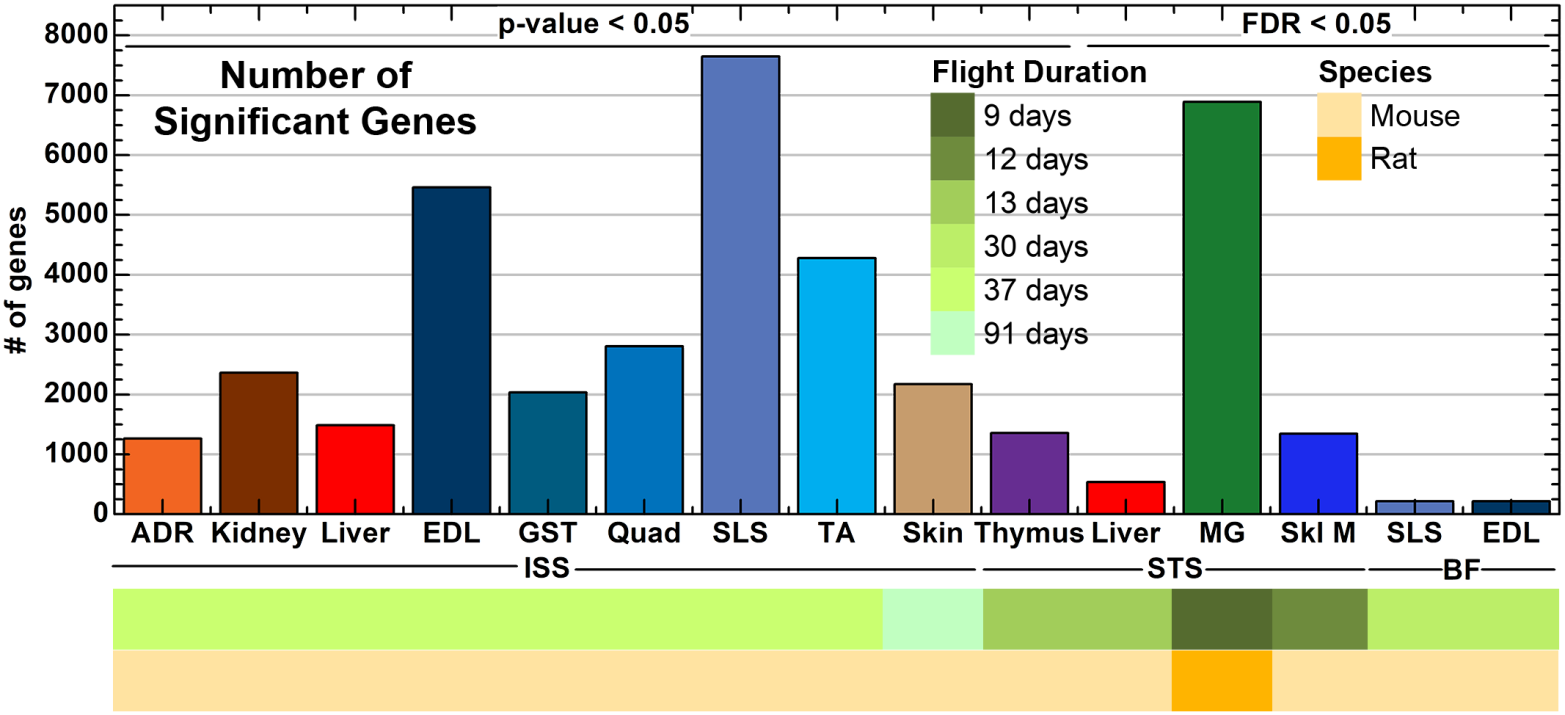
The number of statistically significant genes determined for each tissue and dataset. A bar plot representing the number of significant genes for each tissue from all datasets either determined by t-test with p-value ≤ 0.05 or with FDR ≤ 0.05. The tissues are separated by the flight conditions for the rodents with ISS = International Space Station, STS = Space Shuttle Mission, and BF = Bion. The color-coded bar on the bottom of the plot represents datasets associated with Flight Duration and Rodent Species.

In order to visualize similarity between various animal samples, Principle Component Analysis (PCA) technique was used for each dataset (S1 Fig). PCA results show that skin samples from ISS, the majority of the muscle tissue, and mammary glands from STS had clear separation between the flight and ground Animal Enclosure Module (AEM) controls [22]. When focusing on the largest experimental dataset and the only RNA-seq dataset (i.e. GLDS-48), PCA graph shows strong clustering by tissue type, suggesting tissue type is the driving factor for changes in gene expression (all muscle tissues cluster together—lower left panel, S1 Fig). Even though, separation between flight and ground AEM is not a predominant feature, we still ran statistical test to identify genes that were differentially expressed between both experimental condition for each given tissue and flight condition (see Material and method). We either used a p-value (p<0.05) cutoff for ISS-flown rodents or a FDR adjusted p values (FDR<0.05) for STS and Bion-M1- flown samples to reduce the probability of false positive genes (Fig 2). For each dataset due to the variability in noise and in the number of biological replicates, we used the lowest stringency for the statistics to produce a reasonable number of significant genes and the best overlap between each dataset. For example, in the STS-135 liver samples, STS-70 mammary gland samples, BF SLS and EDL samples, and STS-108 skeletal muscle samples, a first pass at the data using p-value < 0.05 as a cutoff for significant genes yielded a large amount of genes: i.e. 17,168 genes, 10,729 genes, 6,801 genes and 9,087 genes respectively. In contrast, dataset using FDR statistical significant cutoff < 0.05 led to a lower number of genes suggesting this approach was more stringent statistically and reduced chances of getting false positives. With this approach, we showed that, overall a higher number of genes were significantly upregulated in livers of ISS flown mice compared to the STS flown mice. While in the ISS- flown mice, the muscles—Soleus, extensor digitorum longus and tibialis anterior, displayed the highest number of significant genes, in the STS dataset, the highest number of up-regulated genes was found in mammary glands (Fig 2). Interestingly the mammary gland tissue was the only tissue from STS flown rats, while all other tissue was from mice. Muscle tissues from Bion-M1 animals displayed a significantly lower number of upregulated genes than the same tissues from ISS.

### Determination of pan-tissue ‘master regulators’ in flight vs ground AEM studies

**Fig 3:**
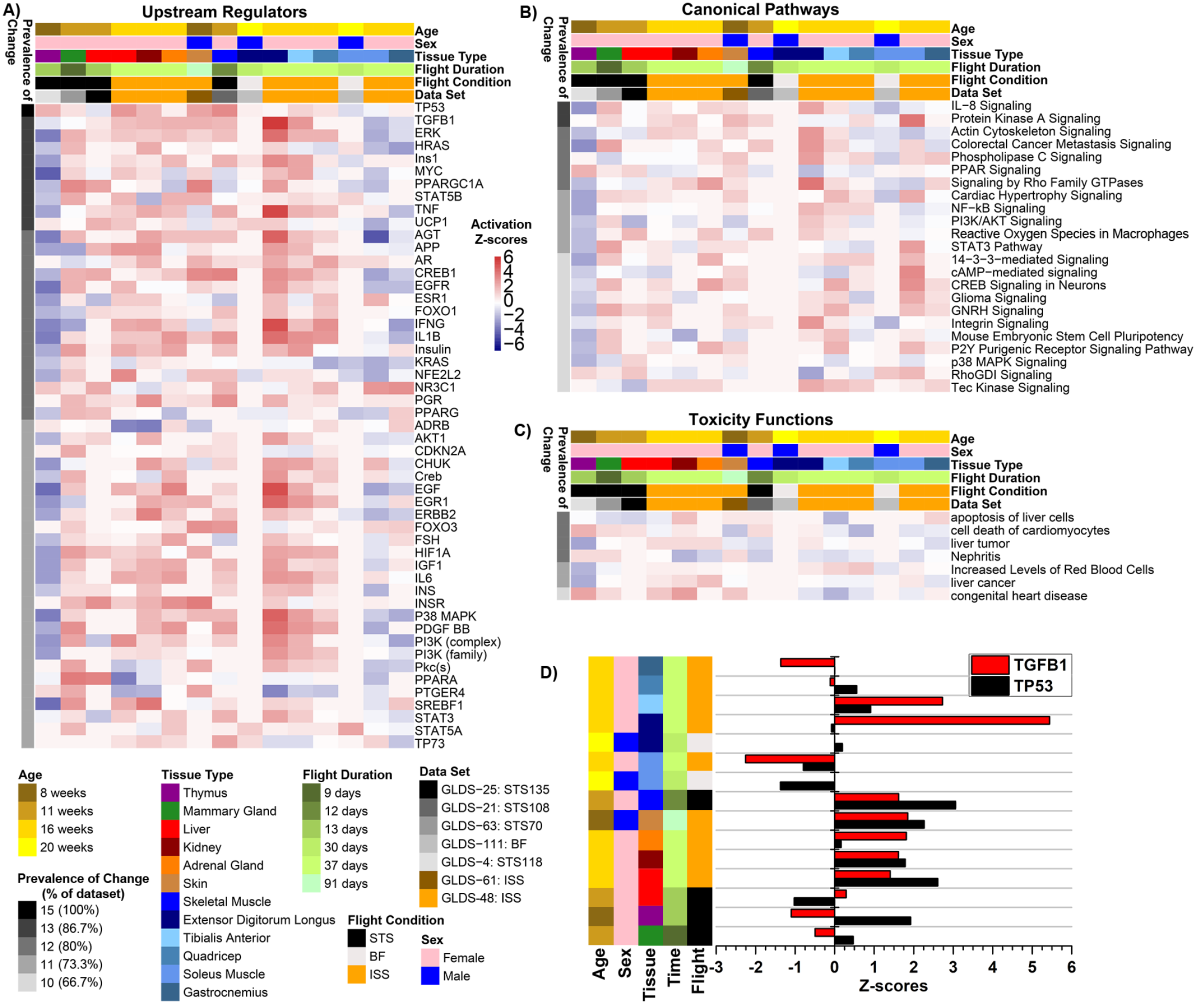
Predicted functions and upstream regulators affected by microgravity. The predicted statistically significant upstream regulators (**A**), canonical pathways (**B**), and toxicity functions (**C**) determined through IPA from data for each individual tissue/dataset using activation Z-score statistics. Heat map representation of the activation Z-score values (red = positive activation Z-score for activation and blue = negative activation Z-score for inhibition) were used to display the data. The prevalence of change (or % of dataset) on the left side of the heat maps represents how common that factor is throughout all datasets/tissues with the darkest color representing factors with the highest degree in common. Age, sex, tissue type, time in flight, flight conditions, and gene lab dataset reference is color coded on the top of the heat maps. For the upstream regulators (**A**) each major cluster of upstream regulators is further analyzed for the major functions it will impact represented by the Resulting Pathways. **D)** A bar graph representing predicted activity of *TGF-β1 and TP53* through z-score statistics comparing tissue type, time of flight, and flight conditions, age and sex of the animals.

In order to gain a comprehensive understanding of how microgravity affected the various rodent datasets, we first isolated the different factors that varied in the datasets—age, sex, duration of flight, flight condition (ISS, STS or BF), tissue type and the dataset in question. Using Ingenuity Pathway Analysis (IPA—QIAGEN Bioinformatics) and the significantly differentially-expressed genes reported in Fig 2, we predicted (based on activation Z-score statistics) prevalence of change in upstream regulators across all the datasets along with the corresponding pathways that were affected per cluster of upstream regulators (Fig 3A). Upstream regulators are molecular factors (enzymes, kinases, transcriptional regulators, cytokines, growth factors, etc.) which act as central hubs involved with numerous significantly differentially-expressed genes [18]. The Prevalence of Change indicates the percentage of datasets in which a specific upstream regulator (Fig 3A), a canonical pathway (Fig 3B), or a toxicity function (Fig 3C) was predicted to be either activated or inhibited from the list of differentially-expressed gene for each dataset. We determined factors included in the Prevalence of Change with either an activation Z-score >0 or <0. For all three analysis we did not find any clustering for activation or inhibition which correlated with experimental factors (e.g. age, flight duration, sex, tissue type). Among the upstream regulators, *TP53* had 100% prevalence of change (Fig 3A) in response to microgravity, while *TGF-β1*, *UCP1*, *STAT5B*, *Ins1*, *HRAS*, *MYC*, *ERK*, *TNF* and *PPARGC1A* showed significant activation/inhibition in 87% of the dataset (Fig 3A). Among the impacted pathways, immune system and inflammation-related and TGF-β1 mediated pathways were the most prevalent ones (Fig 3A and 3B). Interestingly, mild liver toxicity was suggested, with alterations in the pathways “apoptosis of liver cells” and “liver tumor”, along with changes in the pathways associated with “nephritis” and “cell death of cardiomyocytes” (Fig 3C).

The predicted factors, *TP53*, *TGF-β1*, *ERK*, *HRAS*, *Ins1*, *MYC*, *PPARGC1A*, *STAT5B*, *TNF* and *UCP1*, were affected by microgravity and exerted an overall global impact on all tissues, and hence were considered as master regulators. Specifically, *TP53* and *TGF-β1* were predicted to have modestly high activity in skeletal muscle (STS), liver, kidney and skin of ISS flown animals. While the highest expression of *TGF-β1* was observed in EDL, TA and ADR from ISS flown animals *TP53* was relatively highly expressed in the STS derived skeletal muscle (Fig 3D). It is interesting to note in this last figure the high correlation between *TP53* and *TGF-β1* activation levels across the various experiments, suggesting some synergism between both regulators.

**Fig 4:**
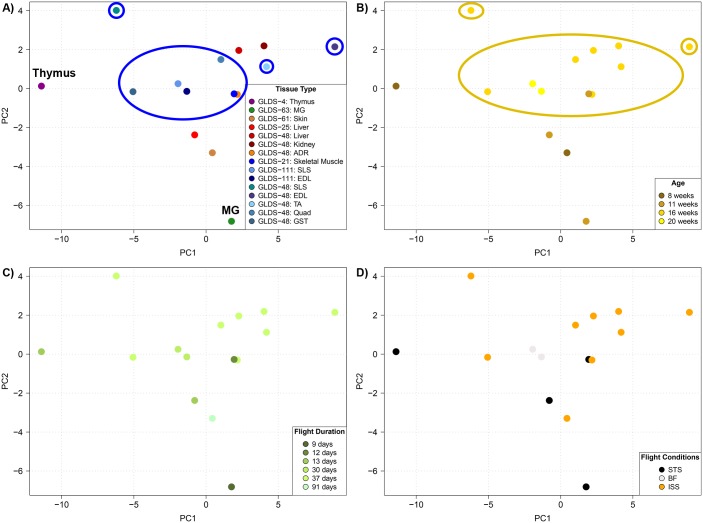
Global clustering of the upstream regulators for all tissue types. Principle Component Analysis (PCA) on the upstream regulators for each **A)** tissue type, **B)** age, **C)** flight duration, and **D)** flight condition. In **A)** the Thymus and Mammary Gland (MG) are specifically labeled for clarity in and the muscle data points are circles in blue. In **B)** a dark yellow circles all date points representing ≥ 16 weeks of age.

Principal Component Analysis technique was used once more on the predicted activity of the upstream regulators this time to capture similarity between the different datasets. In contrast to PCA of gene expressions (S1 Fig) which could only be done for the same dataset, activation Z-score for upstream regulators are platform independent and generate a set of vectors for each dataset that can be plotted simultaneously into one single PCA plot (Fig 4). Doing so, we clearly identified some tissue separating from all other tissues. The most separated one was Thymus, which had been reported during the study to show some degree of atrophy with significant decrease in leukocyte populations, higher DNA fragmentation, and modulation of expression of 15 cancer-related genes and 6 T-cell related genes [10]. Mammary gland (MG) from rats also showed a strong separation at the upstream regulation level. Not as obvious was the cluster of all muscle tissues which separated based on the second principal component PC2 (vertical axis in Fig 4A). It was also revealed that there is a slight age dependence with older mice (≥ 16 weeks) grouping closer together (Fig 4B). This indicates that the overall age of the rodents used has a slight impact on the systemic biological response to microgravity. Changes in predicted activity of the upstream regulators on all the tissues seem to be sex, flight conditions and duration of flight independent (Fig 4C and 4D).

**Fig 5:**
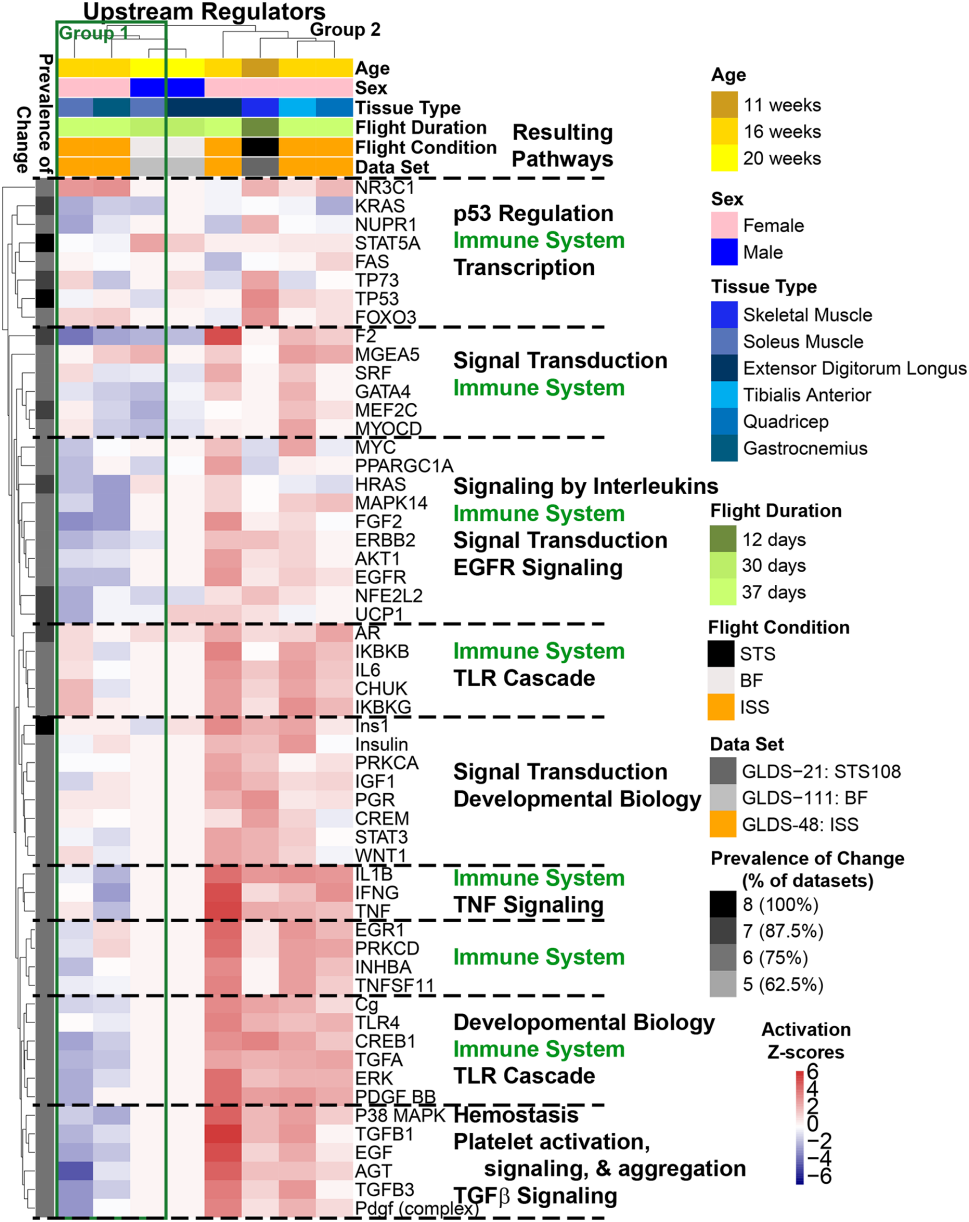
Predicted upstream regulators for muscle tissue affected by microgravity. The statistically significant predicted upstream regulators determined by IPA from data for each individual tissue/dataset using activation Z-score statistics. Heat map representation with hierarchical clustering of the activation Z-score values (red = positive activation Z-score for activation and blue = negative activation Z-score for inhibition) were used to display the data. The prevalence of change (or % of dataset) on the left side of the heat maps represents how common that factor is throughout all datasets/tissues with the darkest color representing factors with the highest degree in common. Age, sex, tissue type, time in flight, flight conditions, and gene lab dataset reference is color coded on the top of the heat maps. Each major cluster of upstream regulators is further analyzed for the major functions it will impact represented by the Resulting Pathways. The green box represents the division between group 1 of muscles (consisting of Soleus and Gastrocnemius) and group 2 (consisting of Extensor Digitorum Longus, Tibialis Anterior, and Quadriceps) having an overall opposite regulation for most of the upstream regulators.

Focusing on the muscle tissue cluster identified in Fig 4A (all showing up inside or near the PC2 positive half), one can note that individual tissue samples have a large spread along the first component in the PCA plot (PC1 –horizontal axis), suggesting different biological processes are at play depending on the muscle type along this component. Performing hierarchical clustering analysis for the activation Z-scores of the upstream regulators in muscle tissue alone, muscle types naturally divided into two sub groups: Gastrocnemius and *Soleus* (“Group 1”, green-bordered box in Fig 5) which displayed similar patterns of predicted altered activity for the majority of the upstream regulators, and Extensor Digitorum Longus, Quadriceps, Tibialis Anterior and Skeletal Muscle (“Group 2”) which consistently had opposite patterns from Group 1 (Fig 5). The two groups of muscle tissues displayed significant differences in the level of predicted activity of crucial signaling genes such as *TNF*, *TGF-β1*, *TGFα*, *p38MAPK*, *ERK*, and *IL1β*. We also found that the predicted activity of PPARGC-1α was highly downregulated in Group 1 muscle and upregulated in all but one of the types in Group 2. This could be due to potential compensatory mechanisms occurring in certain muscle types of the musculoskeletal system as a result of microgravity exposure. By looking at the anatomy of a mouse muscle (S2 Fig), one could interpret these results as an unloading of the muscles in Group 1 not supporting the weight of the animal in microgravity anymore, while muscles in Group 2 would see an increased loading due to pull and grabbing to the cage. Interestingly, microgravity responsive changes in immune-related pathway regulation were observed to be the most prevalent in the muscle Group 1.

### Determination of key microgravity responsive genes and the most connected gene(s)

**Fig 6:**
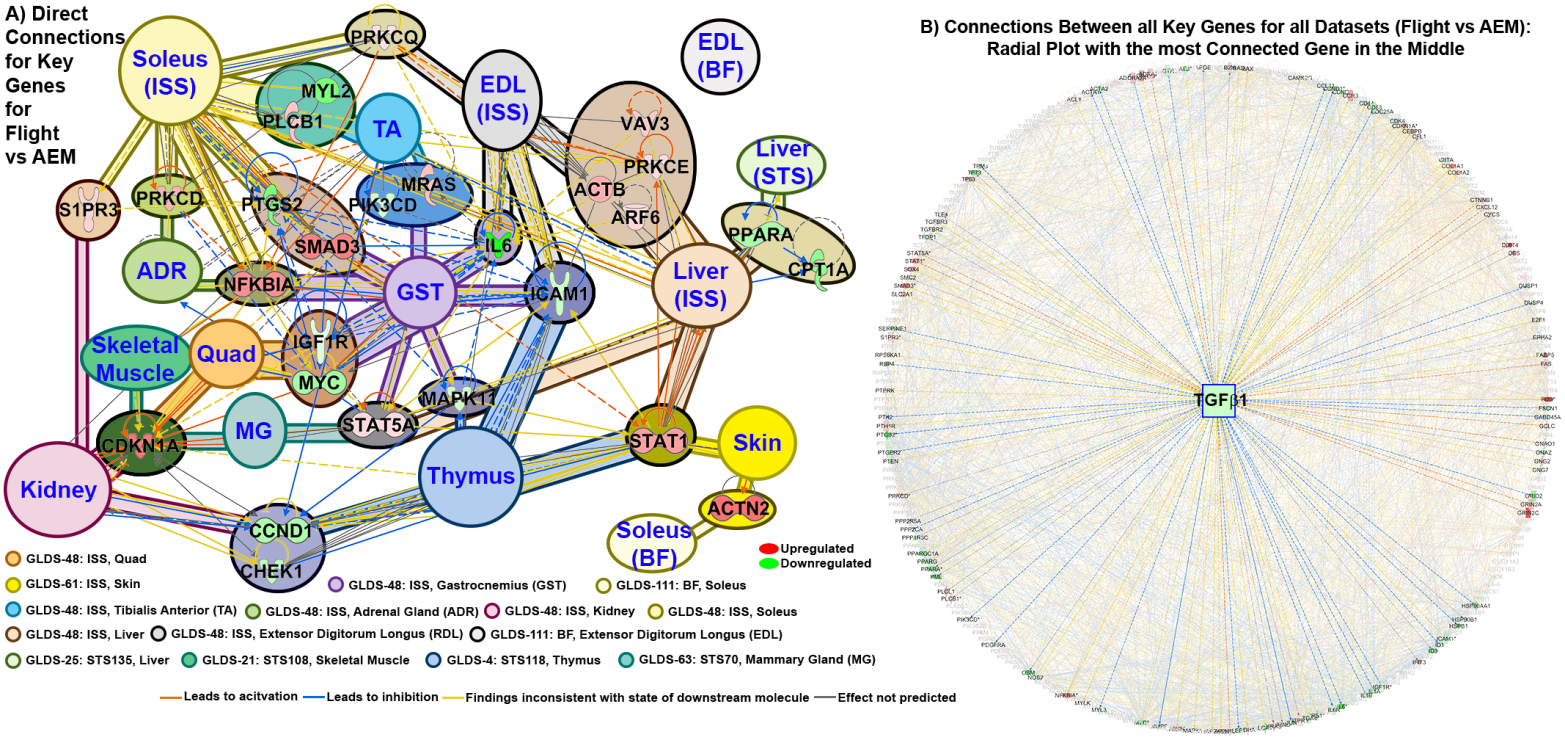
Key genes and master regulators driving pan-tissue microgravity response. **A)** A connectivity network for each set of key genes determined independently for each tissue/dataset. The key genes associated with each individual tissue/dataset are represented by different background colors as indicated in the legend. Overlapping key genes (blue font) between each tissue type is represented by background colors and is considered as central nodes. The cluster of key genes for each dataset that are not connected are grouped and shown as one circle. Details for each of these clusters of key genes for each dataset can be seen in S2 Fig. The color of the gene represents whether the gene is upregulated (red) or downregulated (green) with the shade signifying the degree of regulation. The different line colors represent the predicted effect of each gene on each other. **B)** A radial plot of all connections between all key genes with the most connected gene displayed in the middle (TGFβ1). The key genes with direct connections to TGFβ1 are shown with all other connections shown with faded color.

To determine key genes that impacted the physiological and biochemical processes in rodents exposed to microgravity, we used a system biology approach we previously introduced [7, 9, 21]. Briefly, this method combines statistical tools contained in Gene Set Enrichment Analysis [19] and IPA (detailed description available in methods) to identify the “key genes” driving the observed differentially-expressed genes. We hypothesize, these “key genes” are driving the biological response to spaceflight conditions. To determine if any commonality exists between the sets of key genes for each dataset, we diagrammed connectivity between each dataset to show that a significant number of key genes are shared (Fig 6A and S3 Fig). The specific connections between the key genes associated with each dataset can be thought of central hubs (Fig 6A). The details for the rest of the key genes for each dataset can be found in S3 Fig. These key genes that are shared between the datasets are hypothesized to have the highest global impact in the host affecting multiple tissues, in response to microgravity.

Note that Fig 6A identified *CDKN1A*, *IL6*, *ICAM* and *MAPK11* as key genes, which have common immune pathways and have previously been shown to be impacted by microgravity across various tissues [23, 24]. Similarly, Thymus isolated from STS-118 mice also showed downregulation of expression in several of the signaling nodes such as *CCND1*, *TGF-β1*, and *MYC* as previously shown [23]. The most connected key gene/driver in all tissues (for studies comparing Flight versus ground AEM) was *TGF-β1* (Fig 6B). Transforming growth factor beta (TGF-β) is a pleiotropic cytokine; known to play a context specific role in sustaining tissue homeostasis predominantly via transcriptional regulation of genes involved in differentiation, cell motility, proliferation, cell survival along with regulating immune responses during homeostasis and infection [25].

In an *in vitro* study by Blaber et al. [26], the effects of 15 days of microgravity were assessed on early lineage commitment of mouse embryonic stem cells (mESCs) using the embryoid body (EB) model of tissue differentiation. Although no significant changes were observed in TRP53 in EBs differentiated in microgravity, a few apoptosis related genes such as *BCL2*, *CUL9*, *FADD* and *CASP9* were found to be upregulated in EBs grown at 1g relative to the undifferentiated mESCs. A few p53 target genes such as *STAT1*, *JUN*, and *EGR1* were also significantly down regulated in microgravity-differentiated EBs, relative to 1g control EBs [26]. We compared the overlapping key genes (Fig 6 and S3 Fig) from our analysis (comparing gene expression values) with the genes identified in the microgravity induced EB differentiation and categorized the genes based on their functions (S4 Fig). The full list of these genes and associated categories/pathways are found in Blaber et al [26]. Although *in vitro* conditions may not accurately replicate those *in vivo*, we found similar trends in regulation of several genes in the functional categories of ‘apoptosis’ and ‘cell cycle regulation’. *BIRC5*, *CASP9*, *SFN* and *TNF* involved in apoptosis and *CDC25A* and *KRAS* involved in cell cycle regulation varied consistently between the two studies. The expression of key genes derived from our analysis of *in vivo* flight data, such as *IL6*, *STAT1*, *PPARG/A* and *CCND1/2* (Fig 6) also varied consistently in our study and in Blaber et al. study, suggesting that *in vivo* pan-tissue analysis of gene expression can be used to confirm knowledge about spaceflight related biological effects from *in vitro* studies.

### Delineating the spaceflight-induced circulating miRNA signature and global health risk assessment

**Fig 7:**
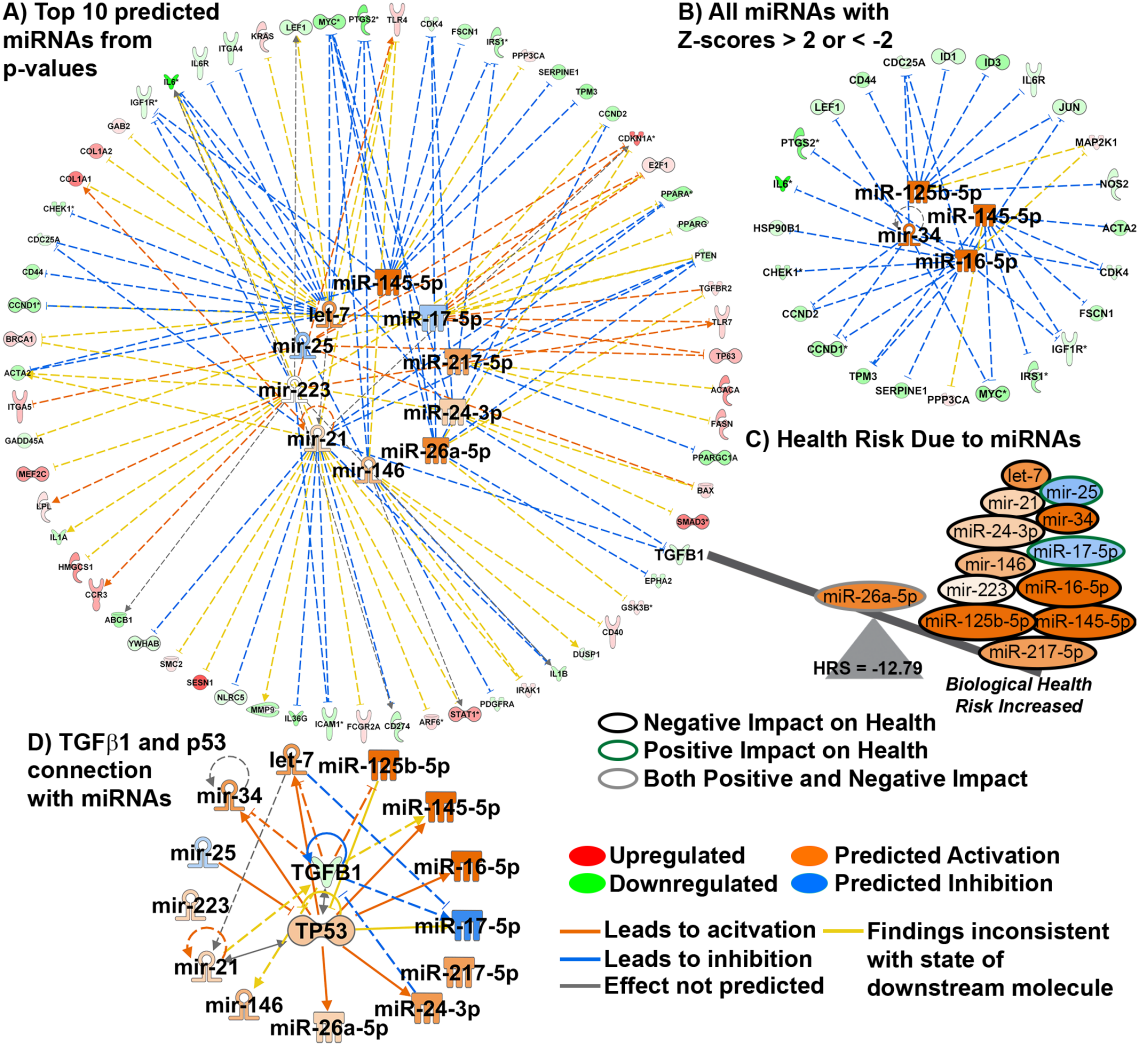
A microgravity associated circulating miRNA signature. **A)** A radial plot showing the top 10 predicted miRNAs with p-values ≤ 1.17 × 10^−8^ determined from all key genes and the key genes directly related the miRNAs. **B)** All statistical significant miRNAs predicted from all key genes with activation Z-score ≥ 2 or ≤ -2 and the corresponding key genes associated with these miRNAs. The predicted activity of each miRNA (blue = inhibition and orange = activation) was determined through activation Z-score statistics through IPA. **C)** A graphical representation of the health risk score (HRS) illustrating how each miRNA contributes to the calculation. The outline for each miRNA represents if the miRNA has a negative impact on health (black), positive impact on health (olive), and has both positive and negative impact on health (grey). **D)** Radial plot connecting TGFβ1 with p53 and all miRNAs. Through activation Z-score statistics in IPA it was determined that p53 will be activated due to the impact of TGFβ1 and the miRNAs.

Examination of a common circulating factor that could connect the TGF-β1 led myriad signaling pathways coordinating systemic response to spaceflight, was warranted given the influence of microRNAs (miRNAs) on TGF-β and p53. MiRNAs are endogenous small noncoding RNAs that each can target hundreds of mRNAs (also protein and DNA) and function as post-transcriptional modulators of gene expression, leading to dysregulation of protein expression and/or mRNA levels [11, 27, 28]. Simulated microgravity study detected elevated levels of miR-223 with decreased proliferation of Hepa1-6 cells [29]. Consistent with this report, our analyses also predicted miR-223 among the top 10 spaceflight-induced miRNAs, which potentially could result in down regulation of several key genes such as *ICAM1*, *IGF1R* and upregulation of *TLR4/7*, *LPL and CCR3* etc. (Fig 7A and S1 Table). In another report, modeled microgravity was found to cause significant overexpression of miR-34a in human lymphoblastoid cells [30]. Our analyses also detected miR-34 to be significantly activated in response to spaceflight, which is involved in potential downregulation of several key genes such as *CCND2/1*, *MYC*, *CDC25a* and *LEF1* (Fig 7B). Compared to normal gravity, in-silico analysis of stimulated human leukocytes showed that brief exposure to spaceflight onboard ISS caused suppression of miR-21 [31]. The same miRNA was predicted in our analyses to cause downregulation of critical key genes such as *TGF-β1*, *PPARA*, *PTEN*, *CDC25a*, *NRLC5*, *ICAM1* and *ILIB* and activation of *COL1A1* (Fig 7A). Using the list of predicted miRNAs a biological Health Risk Score (HRS) was calculated. We have previously used a similar method to calculate a Cancer Risk Score [11]. This HRS provides us with a comprehensive idea about how a group of spaceflight responsive miRNAs could weigh on overall health, positive for lower health risk and negative for higher health risk (Fig 7C). miR-25 and miR-17-5p which have positive health risk showed a predicted inhibition (blue color) leading to a negative impact on health. All the other predicted miRNAs are activated and are known to have a negative impact on health [32–34]. MiR-26a-5p is shown in Fig 7C to have both positive and negative impact on heath. Overall, these spaceflight studies suggest microgravity onboard ISS, STS and Bion-M1 have a strong negative impact on rodent health, with an HRS score of -12.79.

Fig 7D shows the direct interaction of the majority of the predicted microgravity associated miRNAs with the master regulators TGF-β and p53. For example, TGF-β and p53 have been previously implicated in biogenesis of miRNAs including miR-215, which may decrease cell division [28]. TGF-β can function to increase maturation of miR-21, which was shown to inhibit PTEN and Sprouty 1, the crucial negative regulators of the Akt and Ras/MAPK pathways, thus leading to tumor progression [35, 36]. MiR-34 has been found to inhibit TP53 through directly targeting its mRNA [37].

## Conclusions

The analyses presented here demonstrate the utility of publicly available repositories like NASA’s GeneLab to generate hypotheses on the biological impact of microgravity. We found that, irrespective of rodent type, age, sex, flight condition and time of flight, several master regulators coordinated major systemic responses towards microgravity. *TP53*, *TGF-β1* and immune associated signaling were identified to be the most prevalent pan-tissue signaling nodes activated in response to microgravity along with *TGF-β1* being the most connected gene across all datasets.

Transforming growth factor beta (TGF-β) is a pleiotropic cytokine, belonging to a family, which consists of 33 members, including the activins, inhibins, bone morphogenetic proteins (BMPs) and growth and differentiation factors. TGF-β is known to play a context-specific role in sustaining tissue homeostasis predominantly via transcriptional regulation of genes involved in differentiation, cell motility, proliferation, and cell survival along with regulating immune responses during homeostasis and infection. Several previous reports have indicated the modulation of TGF-β gene expression with microgravity. For example, reduction in gravitational force was found to diminish TGF-β expression and apoptosis with higher carcinoembryonic antigen expression in 3D human colorectal carcinoma cells, as compared to 3D cultures in unit gravity [38]. In another study, differential regulation of blood vessel growth by TGF-β using basic fibroblast growth factor was identified in modeled microgravity with induction of early and late apoptosis, extracellular matrix proteins, endothelin-1 and TGF-β1 expression [39]. Bone development involves dynamic remodeling involving gravity-regulated mechanical stimulation for conservation of mineral content and structure. TGF-β has been implicated to function as an autocrine and paracrine regulator of bone formation. Human fetal osteoblastic (hFOB) cells grown in space have been shown to exhibit significantly reduced TGF-β1 and TGF-β2 transcript levels, 24 hours post-flight [40]. Among *in vivo* studies, both short term and long-term flight data report changes in TGF-β mRNA levels in response to weightlessness and microgravity. During an 11-day space mission, rat skeletal muscle exhibited reduced TGF-β expression [41], and a 91-day mission aboard the ISS revealed lower TGF-β expression in colonic tissue, systemic lymph node, inguinal and brachial lymph nodes [42].

Microgravity is one possible cause changing TGF-β expression levels. However, ionizing radiation found in space can also have impact on TGF-β expression levels and the overall biology of organisms in space [43, 44]. For example, upregulation of TGF-β following ionizing radiation may improve DNA repair [45, 46] but it can also elicit a stem-cell self-renewal signaling in breast cells which has been correlated to increased breast cancer risk in young women exposed to ionizing radiation [47]. In addition, our results show that the space environment is globally down-regulating TGF-β1 in the rodent tissue (Figs 6 and 7), which may contribute to additional DNA damage to the host due to HZE irradiation but which may also lower certain cancer risk. These results also suggest that microgravity is the predominant factor affecting TGF-β, leading to lower expression levels in contradiction from what one expects from exposure to ionizing radiation.

TGF-β signaling has also been known to crosstalk with the second key regulator found in this study: i.e. p53 [28]. p53 is a transcription factor and in response to genotoxic stress, DNA damage, oncogene activation, and hypoxia, it is recruited to specific sites in chromatin, promoting transcription of apoptosis related genes [48]. Again, the relationship with TGF-β is complex as a report showed that inactivation of p53 can also alter TGF-β signaling, which ironically displayed both tumor-suppressive and pro-oncogenic functions [49].

Finally, we hypothesized that the global systemic response to microgravity in rodents, driven by TGF-β1 is arbitrated by a circulating miRNA signature consisting of thirteen miRNAs predicted from the key genes. Using the miRNAs functional state and impact on health, we calculated a theoretical “health risk score”. The Health Risk Score is based on known association of this miRNA with reported health outcomes, circumventing the challenging interpretation for health effects resulting from the complex interaction of TGF-β/p53/Immune signaling discussed previously. With this novel approach, we predict that short and long-term space missions exert a strong negative impact on rodent health. Suprisingly the miRNAs that we predict to drive microgravity biological response have been reported to be involved with simulated microgravitry experiments previously reported by other investigators. For example, modeled microgravity-triggered miRNAs have been identified in human peripheral blood lymphocytes (PBL) [27, 50], human leukocytes [31], human lymphoblastoid cells [30], and in murine hepatoma cell line [29]. In these studies, miR-223 has been found to regulate cell cycle progression by targeting E2F1, Fbxw7/Cdc4, IGF1R, Cdk2 and TOX and overexpression of the miRNA resulting in suppression of c-Myc expression [51]. Human peripheral blood lymphocytes (PBLs) cultured for 24 h in microgravity with respect to 1 g, revealed dysregulation of 42 miRNAs, of which miR-34a-5p, miR-34b-5p, and let-7i-3p were found to be in common with our results. Most of the identified miRNAs were correlated with controlling immune-related (TCR signaling, adaptive and innate immune signaling and cytokine signaling), apoptosis and cell proliferation related gene expression.

Several clinical analyses have shown that circulating miRNAs are stable in serum and plasma, are water-soluble, and are easily detectable. Given their modulation of expression profile based on physiological and pathological conditions, their role as therapeutics biomarkers is being investigated [52]. Recent reports on regulation of cell-cell communication by exosomes have led to researchers to investigate further the role of exosome-derived miRNA that can function in the target cells in diverse biological processes [53]. Other than microgravity, acute and chronic levels of ionizing radiation pose threats to astronaut health in space missions [54]. Radiation induced DNA damage has been studied in detail and changes in miRNA expression was detected both *in vivo* and *in vitro* and based on type of cell, type of radiation and repair time, miRNA levels were found to differentially synchronize p53 activity [55, 56]. Hence, cell-free, circulating miRNA may be a useful minimally non-invasive biomarker for the detection of space related health risk, also to monitor progress of the symptoms, and subsequent response to therapeutic countermeasures.

In conclusion, the current study demonstrates the value of repository data in generating novel hypotheses through in-silico analysis. This study revealed microgravity-induced critical genes, signaling pathways and circulating miRNA signatures that may be leveraged for identification of space related health risks biomarkers and in the design of countermeasures for long-term manned missions.

## Supporting information

S1 Fig Global clustering of Flight versus AEM ground controls for each individual dataset. Principle component analysis (PCA) determined from all probes for each dataset comparing Flight versus AEM ground controls.
(TIF)

S2 Fig Schematic of the ventral and medial muscles in a mouse. A schematic depicting the different groups of muscles in the ventral and medial muscles for a mouse. **Muscle Group 1** represents the posterior muscles in the lower leg, including the Gastrocnemius and Soleus, and comprises ~20% of the leg muscle mass. **Muscle Group 2a** represents the anterior muscles of the lower leg, including tibialis anterior and extensor digitorum longus, and comprises of ~10% of the leg muscle mass. **Muscle Group 2b** represents the medial muscles in the upper leg and comprises of 25% of leg muscle mass. **Muscle Group 2c** represents the anterior muscles of the upper leg, including the Quadriceps, and comprises ~20% of the leg muscle mass. Muscle Group 1 is the cluster represented as Group 1 in Fig 5 and all other muscle groups fall in Group 2 in Fig 5.
(TIF)

S3 Fig Detailed network of all key genes and connections between each dataset. A detailed network of the key genes found in Fig 6A for each dataset. Background color for each dataset is provided for each set of key genes. The overlapping key genes are similar to Fig 6A. Each gene is represented by a symbol indicating what type of molecule it is. The color of the gene represents whether the gene is upregulated (red) or downregulated (green) signifying the degree of regulation. The different line colors represent the predicted effect of each gene on each other.
(TIF)

S4 Fig The overlap of all key genes/drivers compared to genes from existing literature. All key genes we determined from all tissues/datasets were compared to the genes impacted by microgravity discussed by Blaber et al [26]. A box plot representation of the fold-change values of the overlapping genes is displayed with our analysis (black) and the values found by Blaber et. al. (red) [26]. The background colors represent the functional category for each group of genes as presented in the Blaber et. al. manuscript [26].
(TIF)

S1 Table Predicted microRNAs (miRNAs) from the all key genes determined through Ingenuity Pathway Analysis (IPA). Activation Z-scores > 0 will predict the miRNA is activated and activation Z-scores < 0 will predict the miRNAs are inhibited. The bold miRNAs are the top miRNAs which were used in our analysis.
(PDF)

S1 Materials and Methods Data from GeneLab platform. (PDF)
