# Supplementary figures and images for: A microRNA signature and TGF-β1 response were identified as the key master regulators for spaceflight response
Source: PLoS One. 2018 Jul 25;13(7):e0199621. doi: 10.1371/journal.pone.0199621 (PMC6059388; doi:10.1371/journal.pone.0199621)

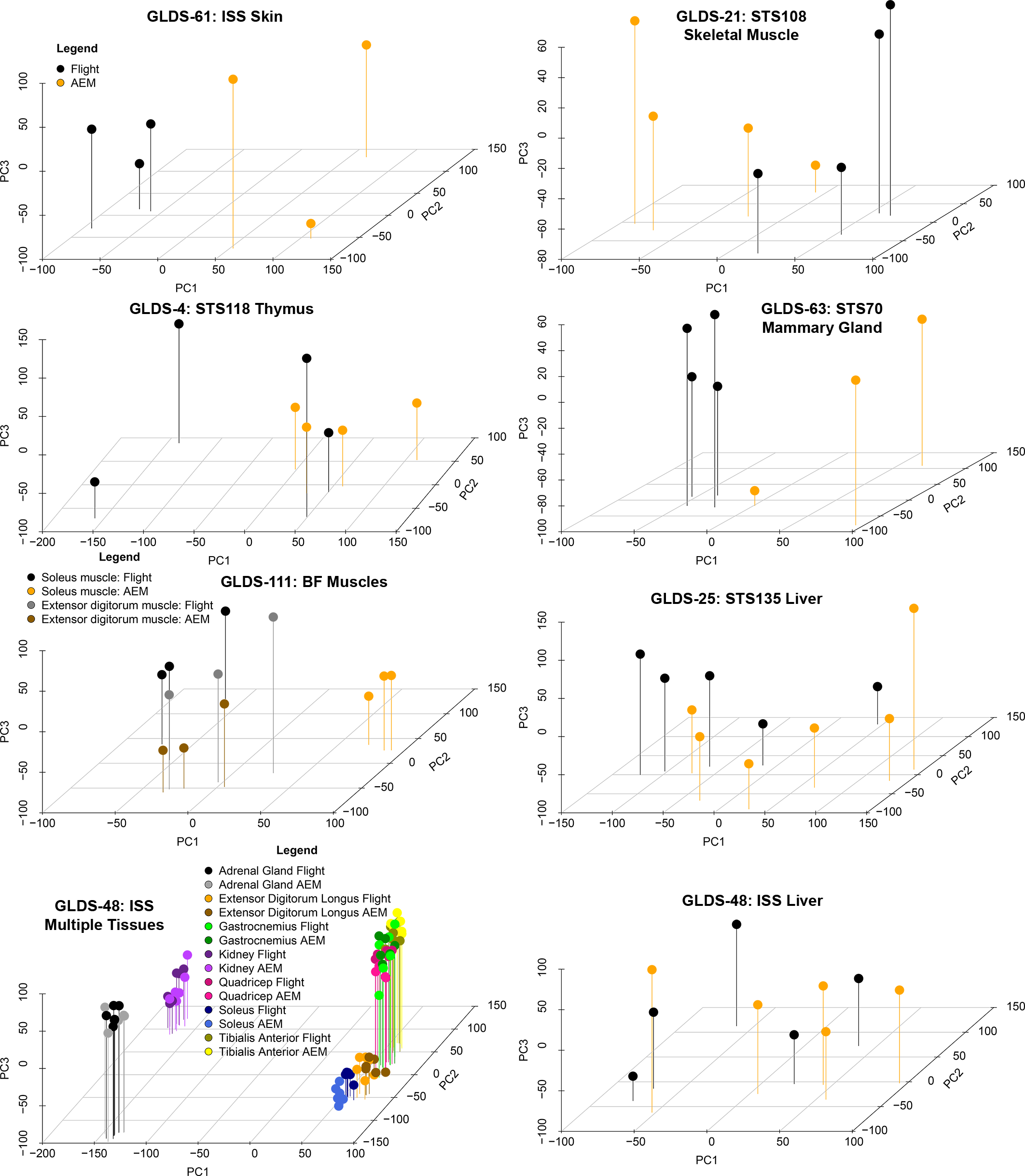

Supplement: S1 Fig — Principle component analysis (PCA) determined from all probes for each dataset comparing Flight versus AEM ground controls. (TIF) [file pone.0199621.s001.tif]

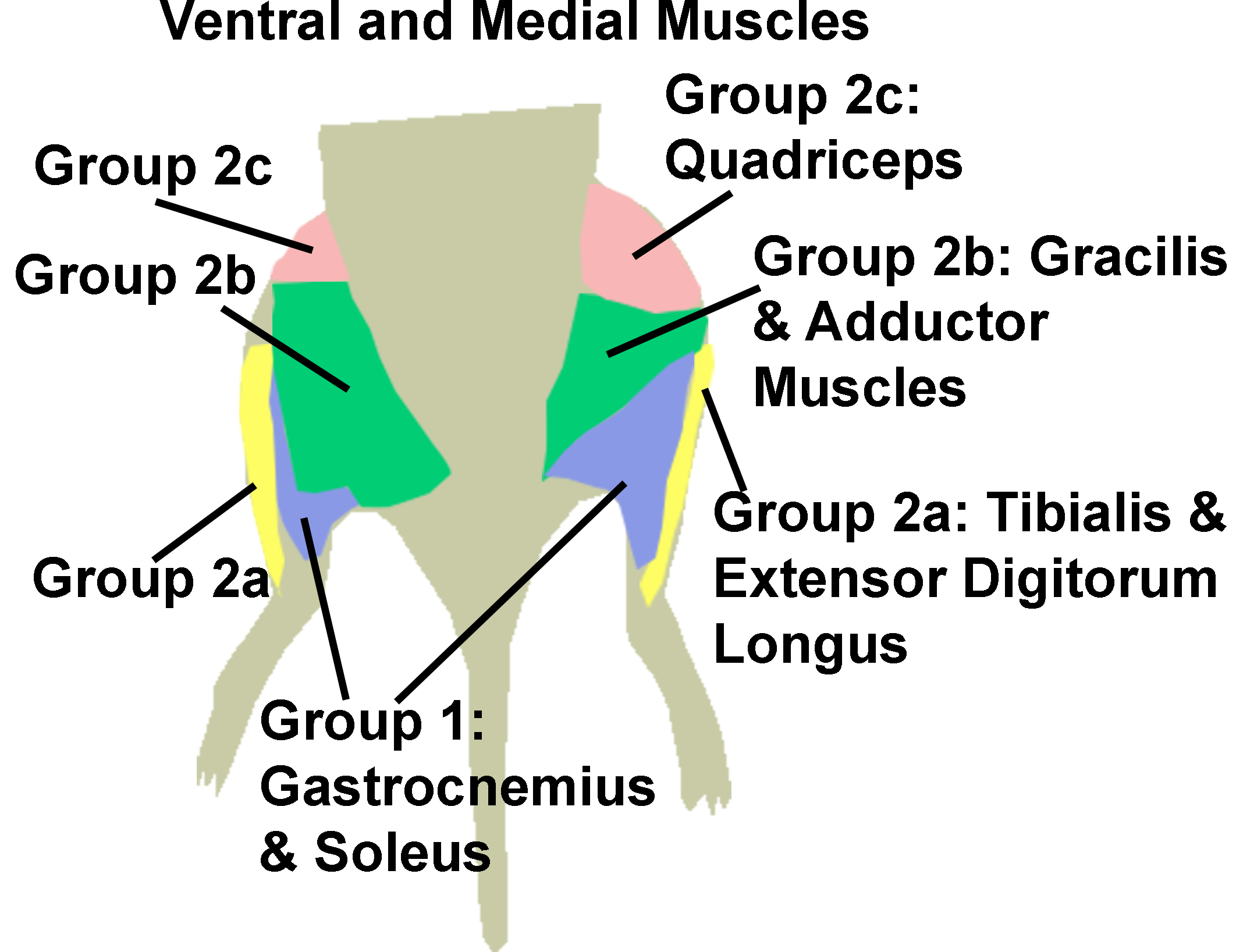

Supplement: S2 Fig — A schematic depicting the different groups of muscles in the ventral and medial muscles for a mouse. Muscle Group 1 represents the posterior muscles in the lower leg, including the Gastrocnemius and Soleus, and comprises ~20% of the leg muscle mass. Muscle Group 2a represents the anterior muscles of the lower leg, including tibialis anterior and extensor digitorum longus, and comprises of ~10% of the leg muscle mass. Muscle Group 2b represents the medial muscles in the upper leg and comprises of 25% of leg muscle mass. Muscle Group 2c represents the anterior muscles of the upper leg, including the Quadriceps, and comprises ~20% of the leg muscle mass. Muscle Group 1 is the cluster represented as Group 1 in Fig 5 and all other muscle groups fall in Group 2 in Fig 5. (TIF) [file pone.0199621.s002.tif]

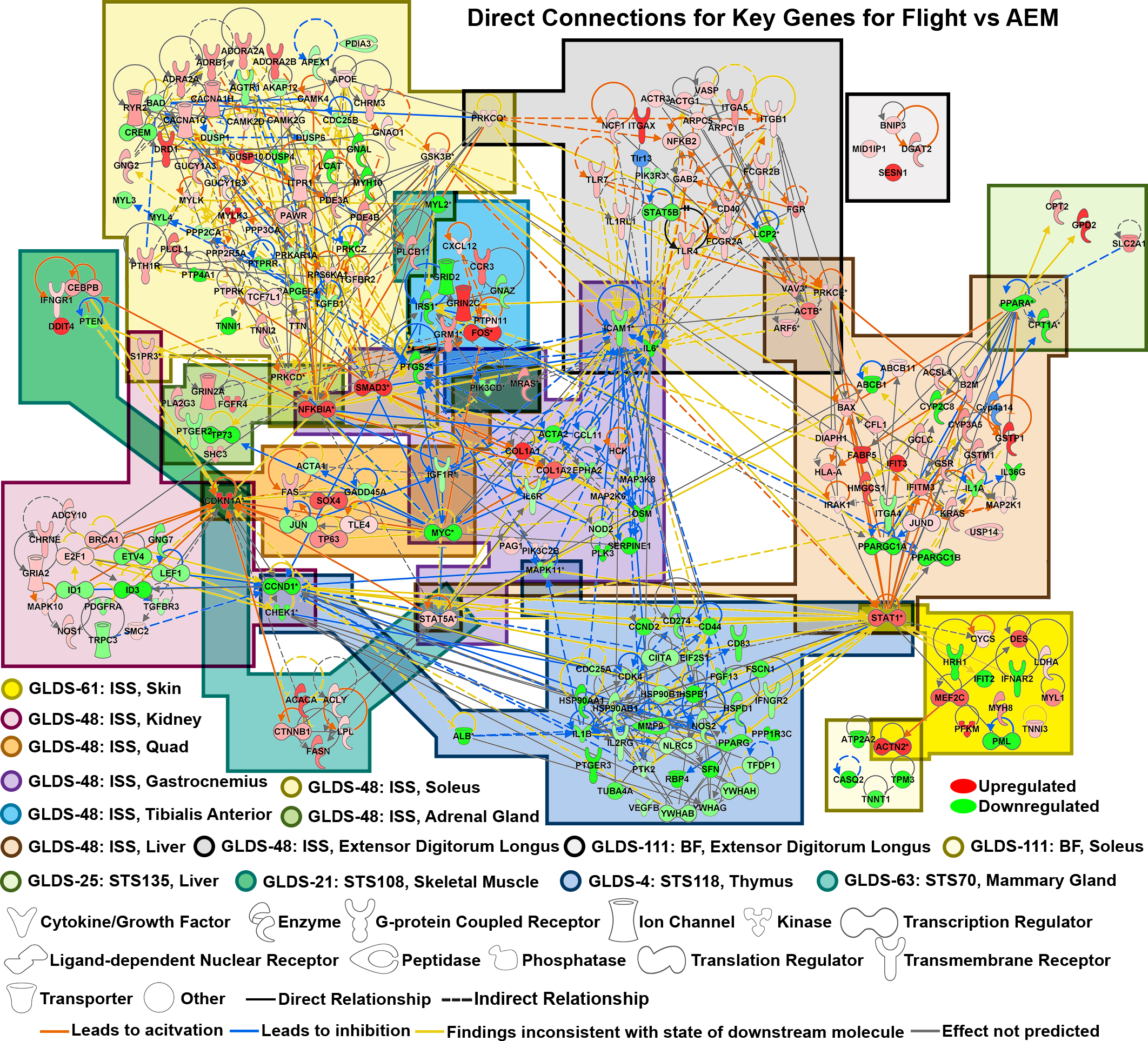

Supplement: S3 Fig — A detailed network of the key genes found in Fig 6A for each dataset. Background color for each dataset is provided for each set of key genes. The overlapping key genes are similar to Fig 6A. Each gene is represented by a symbol indicating what type of molecule it is. The color of the gene represents whether the gene is upregulated (red) or downregulated (green) signifying the degree of regulation. The different line colors represent the predicted effect of each gene on each other. (TIF) [file pone.0199621.s003.tif]

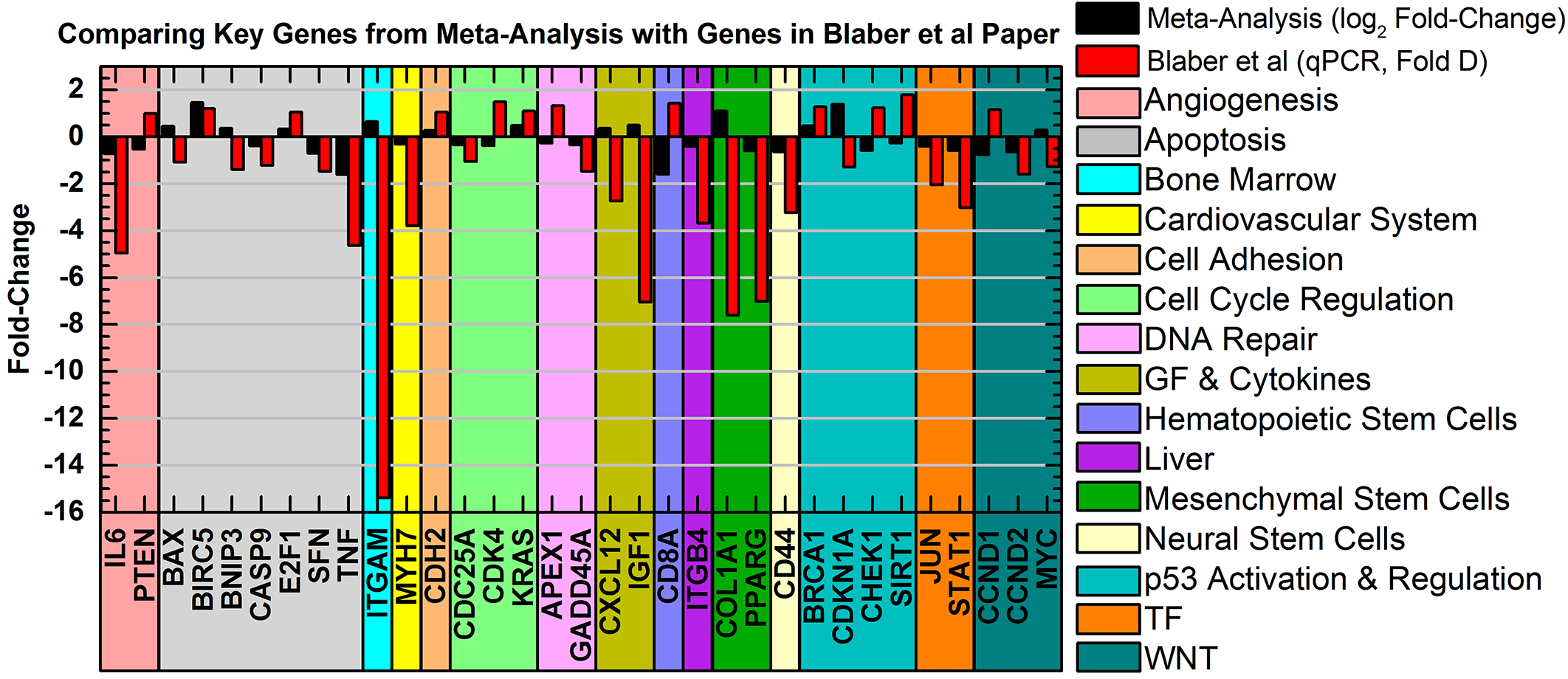

Supplement: S4 Fig — All key genes we determined from all tissues/datasets were compared to the genes impacted by microgravity discussed by Blaber et al [26]. A box plot representation of the fold-change values of the overlapping genes is displayed with our analysis (black) and the values found by Blaber et. al. (red) [26]. The background colors represent the functional category for each group of genes as presented in the Blaber et. al. manuscript [26]. (TIF) [file pone.0199621.s004.tif]
