# Supplementary material for: A microRNA signature and TGF-β1 response were identified as the key master regulators for spaceflight response
Source: PLoS One. 2018 Jul 25;13(7):e0199621. doi: 10.1371/journal.pone.0199621 (PMC6059388; doi:10.1371/journal.pone.0199621)
Supplement: S1 Table — Activation Z-scores > 0 will predict the miRNA is activated and activation Z-scores < 0 will predict the miRNAs are inhibited. The bold miRNAs are the top miRNAs which were used in our analysis. (PDF) [file pone.0199621.s005.pdf]

| Predicted miRNAs | Activation z-score | p-value of overlap | Target molecules in dataset |
|---|---|---|---|
| **miR-16-5p** | **2.728** | **3.40E-05** | **CCND1, CCND2, CDC25A, CHEK1, HSP90B1, IGF1R, IL6, JUN, MAP2K1, PTGS2, TPM3** |
| **mir-34** | **2.392** | **1.82E-05** | **CCND1, CCND2, CDC25A, CDK4, LEF1, MYC** |
| **miR-145-5p** | **2.328** | **2.11E-08** | **ACTA2, CDK4, FSCN1, IGF1R, IRS1, MYC, PPP3CA, SERPINE1, TPM3** |
| **miR-125b-5p** | **2.208** | **1.44E-05** | **CD44, CDC25A, ID1, ID3, IL6R, JUN, MAP2K1, NOS2** |
| **let-7** | **1.106** | **3.01E-13** | **ACTA2, BRCA1, CCND1, CD44, CDC25A, CHEK1, COL1A1, COL1A2, GAB2, IGF1R, IL6, IL6R, ITGA4, KRAS, LEF1, MYC, PTGS2, TLR4** |
| **mir-21** | **0.387** | **4.42E-13** | **ACTA2, ARF6, CD274, CDC25A, CDKN1A, COL1A1, FCGR2A, ICAM1, IL1B, IL36G, IL6, IRAK1, MMP9, NLRC5, PPARA, PTEN, SESN1, SMC2, STAT1, TGFB1, TLR4, YWHAB** |
| **mir-223** | **0.016** | **2.84E-12** | **ABCB1, ACTA2, CCR3, CD274, E2F1, HMGCS1, ICAM1, IGF1R, IL1A, IL1B, IL6, LEF1, LPL, MEF2C, TLR4, TLR7** |
| **miR-26a-5p** | **1.334** | **1.49E-11** | **CCND2, CDK4, CDKN1A, EPHA2, GSK3B, MYC, PTEN, PTGS2, TGFBR2** |
| **mir-146** | **0.669** | **5.95E-11** | **CD40, DUSP1, IL1B, IL6, IRAK1, MYC, PDGFRA, PTGS2, STAT1, TLR4** |
| **mir-25** | **-0.593** | **8.51E-10** | **BAX, CDKN1A, E2F1, GADD45A, IL6, ITGA5, PTEN, TP63** |
| **miR-217-5p** | **0.928** | **1.12E-09** | **ACACA, FASN, IRS1, PPARA, PPARGC1A, PTEN** |
| **miR-17-5p** | **-0.355** | **2.32E-09** | **CCND1, CCND2, CDKN1A, E2F1, PPARA, PPARG, PTEN, TGFBR2, TLR7, TP63** |
| **miR-24-3p** | **0.416** | **1.17E-08** | **BAX, BRCA1, CDK4, IL6, MYC, SMAD3, TGFB1** |
| miR-92a-3p | 0.535 | 2.80E-08 | CCND1, CCND2, CDKN1A, IL6, ITGA5, PTEN, TP63 |
| miR-21-5p | -0.093 | 2.88E-08 | ACTA2, CDC25A, CDKN1A, FAS, IL6R, IRAK1, PTEN, SESN1, TGFBR2 |
| mir-145 | 1.296 | 3.41E-08 | ACTA2, CACNA1C, FSCN1, IL6, IRS1, MYC, PDGFRA, PRKCE |
| miR-155-5p | 0.997 | 5.51E-08 | AGTR1, CCND1, CEBPB, CTNNB1, IL1A, IL1B, IL6, KRAS, LPL, NOS2, PDE3A, PTGS2, SERPINE1 |
| mir-10 | 0.528 | 6.29E-08 | BAX, CD40, CD44, E2F1, GAB2, IGF1R, IL6, IL6R, PPP2CA, RPS6KA1 |
| miR-204-5p | -0.186 | 1.62E-07 | ARPC1B, CDC25B, MMP9, PPARG, PTPN11, SOX4 |
| miR-34a-5p | 0.613 | 1.88E-07 | CCND1, CDK4, CDKN1A, E2F1, ICAM1, MAP2K1, MYC, PPARG |
| miR-141-3p | 0.152 | 2.53E-07 | BAX, COL1A2, CTNNB1, GADD45A, ITGB1, LEF1, STAT5B, TP73 |
| let-7a-5p | 0.96 | 2.64E-07 | ACTA2, CCND1, CCND2, CDC25A, COL1A1, COL1A2, ITGA4, KRAS, MMP9, MYC, PTGS2, TLR4 |
| miR-146a-5p | -1.888 | 1.07E-06 | BRCA1, CCR3, CD40, IL36G, IRAK1, NFKBIA, NOS2, STAT1, TLR4 |
| mir-27 | -1.296 | 2.49E-06 | ABCB1, IL1B, IL6, KRAS, PPARA, PPARG, PTGS2 |
| mir-24 | 0.896 | 3.68E-06 | CDK4, CDKN1A, IL6, MYC, SERPINE1, TGFB1 |
| mir-33 | 0.728 | 3.83E-06 | ACACA, CPT1A, LPL, PPARG, PPARGC1A |
| mir-155 | 0.508 | 3.98E-06 | AGTR1, CCND1, CDKN1A, CEBPB, IL1B, IL6, NOS2, PTGS2, SERPINE1 |
| mir-373 | 0.585 | 8.69E-06 | CD44, IL6, SERPINE1, TGFBR2 |

| | | | |
|---|---|---|---|
| mir-127 | -0.868 | 1.24E-05 | DUSP1, IL1B, IL6, NOS2 |
| mir-15 | 1.128 | 1.26E-05 | CCND1, CCND2, CHEK1, IL1B, IL6, MMP9, PTGS2 |
| mir-515 | 0.739 | 1.72E-05 | CD44, IL6, SERPINE1, TGFBR2 |
| mir-7 | 0.264 | 1.72E-05 | BAX, CDKN1A, IGF1R, PIK3R3 |
| miR-503-5p | 1.188 | 2.33E-05 | CCND1, CDC25A, CDKN1A, CHEK1 |
| miR-18a-5p | -1.982 | 2.33E-05 | CDKN1A, E2F1, KRAS, TP63 |
| mir-192 | 1.082 | 3.99E-05 | ACTA2, CDKN1A, IGF1R, NOD2 |
| mir-1 | 0.524 | 4.57E-05 | CCND2, CEBPB, HSP90B1, HSPD1, IGF1R, KRAS, MMP9, MYLK |
| mir-30 | -0.73 | 4.62E-05 | BAX, CDKN1A, GADD45A, IL6, SERPINE1 |
| mir-143 | -0.246 | 5.08E-05 | KRAS, PDGFRA, PRKCE, PTGS2 |
| mir-17 | -0.148 | 5.57E-05 | CCND1, CDKN1A, E2F1, FAS, LEF1, PTEN |
| miR-23a-3p | 0.192 | 7.89E-05 | CXCL12, IL6R, PTEN, SMAD3 |
| miR-296-5p | 0.132 | 7.89E-05 | ABCB1, BAX, CCND1, COL1A1 |
| mir-19 | -0.94 | 9.66E-05 | CDKN1A, E2F1, PTEN, TP63 |
| miR-27a-3p | -1.103 | 1.03E-04 | ADORA2B, BAX, MEF2C, PPARG, SMAD3 |
| mir-133 | 0.669 | 1.47E-04 | CCND2, FSCN1, GSTP1, IGF1R, PPARG |
| miR-199a-5p | 0.128 | 1.77E-04 | ACTA1, CDKN1A, COL1A1, ID3, TGFBR2, TGFBR3 |
| mir-181 | -0.092 | 1.82E-04 | DUSP6, GRIA2, PRKCD, PTEN, PTPN11 |
| miR-221-3p | 0.153 | 3.10E-04 | DDIT4, FOS, ICAM1, PTEN |
| miR-30c-5p | 0.378 | 3.14E-04 | ATP2A2, GPD2, IL6, JUN, PPARG, PPP3CA, PTPRK |
| mir-8 | 1.619 | 4.79E-04 | CDC25B, CTNNB1, ITGB1, PTEN, YWHAB, YWHAG |
| miR-291a-3p | 0.841 | 6.14E-04 | CCND1, CCND2, CD44, CD83, CDKN1A, TP63 |
| mir-322 | | 8.69E-05 | CDC25A, IGF1R, MAP2K1 |
| miR-451a | | 1.83E-04 | ABCB1, CCND1, MMP9 |
| mir-218 | | 4.25E-04 | LEF1, PDGFRA, VAV3 |
| miR-100-5p | | 5.36E-04 | CCND1, ID1, IGF1R |
| miR-143-3p | | 6.64E-04 | KRAS, PDGFRA, PRKCE |
| mir-221 | | 8.11E-04 | MMP9, PTEN, STAT5A |
| miR-132-3p | | 8.11E-04 | GRIN2A, MMP9, SOX4 |
| miR-515-5p | | 8.19E-04 | PIK3C2B, TCF7L1 |
| miR-193a-5p | | 8.19E-04 | IL2RG, TP73 |
| mir-26 | | 9.76E-04 | CCND2, PTEN, PTGS2 |
| mir-503 | | 1.35E-03 | CDC25A, IGF1R |
| miR-7a-5p | | 1.37E-03 | FOS, IGF1R, IRS1 |
| mir-214 | | 1.37E-03 | IGF1R, PTEN, PTGS2 |
| mir-183 | | 1.59E-03 | IRS1, ITGB1, PTEN |
| mir-103 | | 2.41E-03 | CACNA1C, FASN, PRKCE |
| mir-296 | | 2.80E-03 | COL1A1, IL1B |
| miR-491-5p | | 2.80E-03 | MMP9, PTK2 |
| mir-224 | | 2.80E-03 | CDKN1A, MMP9 |
| miR-140-5p | | 3.70E-03 | CXCL12, SMAD3 |

| | | | |
|---|---|---|---|
| mir-132 | | 4.27E-03 | GRIN2A, PTEN, SOX4 |
| miR-193a-3p | | 4.72E-03 | CCND1, PTK2 |
| mir-194 | | 4.72E-03 | ACTA2, E2F1 |
| mir-101 | | 7.11E-03 | DUSP1, PTGS2 |
| miR-214-3p | | 7.11E-03 | BAX, PTEN |
| miR-96-5p | | 8.46E-03 | IRS1, KRAS |
| miR-223-3p | | 9.92E-03 | IRS1, MEF2C |
| miR-19b-3p | | 9.92E-03 | CCND1, PTEN |
| mir-196 | | 1.15E-02 | COL1A1, FOS |
| mir-130 | | 1.15E-02 | ABCB1, PPARA |
| miR-513a-5p | | 1.18E-02 | CD274 |
| miR-18a-3p | | 1.18E-02 | KRAS |
| miR-545-3p | | 1.18E-02 | BRCA1 |
| miR-574-3p | | 1.18E-02 | TP63 |
| miR-216a-5p | | 1.18E-02 | PTEN |
| miR-330-3p | | 1.18E-02 | E2F1 |
| mir-493 | | 1.18E-02 | IGF1R |
| miR-205-5p | | 1.49E-02 | PRKCE, PTEN |
| mir-205 | | 1.49E-02 | CTNNB1, PRKCE |
| mir-203 | | 1.68E-02 | MMP9, TP63 |
| miR-199a-3p | | 1.68E-02 | CD44, PTGS2 |
| miR-133a-3p | | 2.23E-02 | FSCN1, IGF1R, PTPRK |
| miR-16-1-3p | | 2.35E-02 | CHEK1 |
| miR-511-5p | | 2.35E-02 | TLR4 |
| miR-185-3p | | 2.35E-02 | MYC |
| mir-95 | | 2.35E-02 | BRCA1 |
| mir-485 | | 2.35E-02 | GRIA2 |
| mir-383 | | 2.35E-02 | MYC |
| miR-1298-5p | | 2.35E-02 | PTK2 |
| miR-181a-5p | | 3.23E-02 | GRIA2, KRAS |
| miR-2682-5p | | 3.50E-02 | MYC |
| miR-424-3p | | 3.50E-02 | CHEK1 |
| miR-615-3p | | 3.50E-02 | PPARG |
| miR-370-3p | | 3.50E-02 | MAP3K8 |
| mir-489 | | 3.50E-02 | PTPN11 |
| mir-499 | | 3.50E-02 | PPP3CA |
| miR-125b-1-3p | | 3.50E-02 | IL1B |
| mir-149 | | 3.50E-02 | E2F1 |
| mir-126 | | 3.74E-02 | CXCL12, IRS1 |
| miR-29b-3p | | 3.99E-02 | COL1A1, COL1A2, PTEN |
| mir-22 | | 4.01E-02 | CDKN1A, LEF1 |

| | | | |
|---|---|---|---|
| mir-135 | | 4.01E-02 | CXCL12, IL1B |
| mir-128 | | 4.64E-02 | BAX |
| miR-191-5p | | 4.64E-02 | IL6 |
| miR-125b-2-3p | | 4.64E-02 | BAX |
| mir-574 | | 4.64E-02 | TP63 |
| miR-328-3p | | 4.64E-02 | CD44 |
| miR-149-5p | | 4.64E-02 | E2F1 |
| miR-378a-3p | | 4.64E-02 | IGF1R |
| mir-29 | | 4.81E-02 | COL1A1, LPL, PTEN |

**Supplemental Table 1.** Predicted microRNAs (miRNAs) from the all key genes determined through Ingenuity Pathway Analysis (IPA). Activation Z-scores > 0 will predict the miRNA is activated and activation Z-scores < 0 will predict the miRNAs are inhibited. The bold miRNAs are the top miRNAs which were used in our analysis.
