# Supplementary material for: A microRNA signature and TGF-β1 response were identified as the key master regulators for spaceflight response
Source: PLoS One. 2018 Jul 25;13(7):e0199621. doi: 10.1371/journal.pone.0199621 (PMC6059388; doi:10.1371/journal.pone.0199621)
Supplement: S1 Materials and Methods — (PDF) [file pone.0199621.s006.pdf]

## Supplemental Materials and Methods

### Data from GeneLab Platform

All data used for this manuscript were obtained from GeneLab (genelab.nasa.gov). The following datasets were used: GLDS-25, -21, -63, -111, -4, -61 and 48. Spaceflight mission and experimental details for each dataset such as, the handling of the rodents, tissue processing, RNA extraction and raw data pertaining to either microarray or RNA-sequencing, can be found in the GeneLab database. Briefly, we used 7 different murine and rat datasets for our analysis and examined the following tissues: liver, kidney, adrenal gland, thymus, mammary gland, skin, and skeletal muscle (soleus, extensor digitorum longus, tibialis anterior, quadriceps, and gastrocnemius) (**Fig. 1**). The rodents flown on ISS, STS and Bion shuttles were euthanized either on orbit or on ground, a few hours after landing. Critical organs were harvested and shipped to the laboratories for DNA, RNA and protein isolation and 'omics' datasets were generated. GLDS-25, -21 and -4 datasets were obtained from 9 to 11-week old, female C57BL/6 and C57BL/6NTac mice that were flown onboard on the several Space Transportation systems- STS 108, 118 and 135 and exposed to conditions of microgravity for 12 days and 13 days respectively in either the Animal Enclosure Module (AEM) or the Commercial Biomedical Testing Module (CBTM). These animals were sacrificed within 3-5 hours after landing and the following tissues were collected – liver (GLDS-25), skeletal muscle (Ski M) (GLDS-21) and thymus (GLDS-4). GLDS-63 dataset was derived from female pregnant Sprague-Dawley rats, which were flown aboard the STS-70, for 9 days in the AEM module and sacrificed within 1 h after shuttle landing. Mammary glands (MG) were collected from the rats. GLDS-111 dataset was procured from 19-20 week-old male C57BL/N6 mice that were flown for 30 days in the BION-M1 satellite and sacrificed 12-14 hours after landing. Soleus muscle (SLS) and extensor digitorum longus muscle (EDL) samples were obtained from the animals. On the other hand, GLDS-61 dataset was obtained from 8-week old C57BL/J10 mice, which were housed for 91 days in the Mouse Drawer System (MDS) on the International Space Station (ISS) and sacrificed on orbit. Skin samples were harvested from these animals. Similarly, GLDS-48 sample sets were obtained from 16 week-old, female C57BL/6 mice, which were in orbit for 37 days in the AEM-X habitat and were euthanized on orbit. The following organs were harvested from the carcasses - Soleus muscle (SLS), extensor digitorum longus muscle (EDL), gastrocnemius muscle (GST), quadriceps (Quad), tibialis anterior muscle (TA), adrenal glands (ADR), kidney and liver. For all the above datasets, the ground control mice were housed for the same number of days, in similar AEM habitats with similar diet and were sacrificed to harvest the same organs.
